# Characteristics of LGBTQ+ Patients and Their Care in Comparison with Heterosexual Individuals: What Is Important for the OBGYN?

**DOI:** 10.3390/medicina61071209

**Published:** 2025-07-02

**Authors:** Gabija Didžiokaitė, Paulina Leškevičiūtė, Aida Kuznecovaitė, Virginija Paliulytė

**Affiliations:** 1 Faculty of Medicine, Vilnius University, 01513 Vilnius, Lithuania; paulina.leskeviciute@mf.stud.vu.lt (P.L.); aida.kuznecovaite@mf.stud.vu.lt (A.K.); virginija.paliulyte@mf.vu.lt (V.P.); 2 Department of Pathology and Forensic Medicine, Institute of Biomedical Sciences, Faculty of Medicine, Vilnius University, 03101 Vilnius, Lithuania; 3 Clinic of Obstetrics and Gynaecology, Institute of Clinical Medicine, Faculty of Medicine, Vilnius University, 03101 Vilnius, Lithuania

**Keywords:** LGBTQ+, homosexual, heterosexual, discrimination, lifestyle habits, obstetrics and gynecology, OBGYN, healthcare, risky sexual behavior, prejudice

## Abstract

*Background and Objectives*: Women of the LGBTQ+ community, like heterosexual women, face a wide range of health issues and have a right to comprehensive healthcare. Unfortunately, they often do not seek healthcare due to concerns about possible discrimination or prejudice. The aims of this study were to analyze and compare experiences of LGBTQ+ individuals and heterosexual women during OBGYN appointments in Lithuania as well as to analyze the health of individuals whose biological gender is female and their lifestyle’s effect on their health. *Materials and Methods*: An anonymous online survey was conducted. Respondents answered questions regarding their gender and social identity; obstetrical, gynecological, and general clinical history; sexual life characteristics; and their experiences of visiting OBGYNs in Lithuania. *Results*: This study revealed that some lifestyle habits of LGBTQ+ respondents are more similar to those of heterosexuals than is often hypothesized. However, it also underscored such issues as the more common consumption of psychotropic substances, higher rates of depression, and more prevalent risky sexual practices among the LGBTQ+ community, as well as some neglected topics of OBGYN care in Lithuania. *Conclusions*: This study is the first in Lithuania to analyze the characteristics of LGBTQ+ individuals whose biological sex is female in relation to the field of obstetrics and gynecology. It provides important insights for the further improvement of the healthcare system regarding this topic.

## 1. Introduction

According to the studies of the last decades, LGBTQ+ (Lesbian, Gay, Bisexual, Transgender, Queer and other sexual and gender identities not encompassed in the acronym) community members receive poorer healthcare compared to heterosexual people, are more likely to consume harmful amounts of tobacco and alcohol, and are also more inclined towards obesity [1,2,3]. Discrimination, bullying, and other mistreatment are constant stressors in the lives of most members of the LGBTQ+ community [4]. Heteronormative assumptions are often dominant in the healthcare environment [5]. The OBGYN office is no exception. Healthcare professionals have a unique opportunity to reduce these stressors by providing informed and nonjudgmental healthcare.

Females of the LGBTQ+ community, as well as heterosexual women, may have gynecological problems and various medical questions about their reproductive system, sexual relations, etc. It is important for them to be open with their OBGYN in order to be able to ask the relevant questions and receive individualized care, adjusted to their needs and risks.

In Lithuania, there are both public and private healthcare sectors. In both sectors, consulting with an obstetrician/gynecologist at the primary level is available to all individuals of female biological sex without a referral from a general practitioner or another physician [6]. In the public sector, appointments with an OBGYN are free; however, the wait times are longer, whereas in the private sector, appointments are paid. At the moment, there are no specific individualized care or care protocols for the LGBTQ+ community in the field of obstetrics and gynecology in Lithuania. Therefore, all individuals of female biological sex receive the same standard treatment in both the private and public sectors and are invited to participate in the same preventive programs for the early detection of cervical and breast cancer by their primary healthcare institutions.

Despite the general progress in the field of medicine, there is still a lack of knowledge about LGBTQ+ women’s experiences with healthcare professionals, including OBGYNs, in both the private and public sectors. Therefore, the main aims of this study were to explore and analyze various aspects of the health and lifestyle of individuals whose biological sex is female, focusing on the differences between LGBTQ+ and heterosexual respondents, as well as to analyze and compare the experiences of the LGBTQ+ community and heterosexual women during OBGYN appointments in Lithuania in order to identify the challenges that individuals with female sex may face regarding their healthcare in the field of obstetrics and gynecology. By investigating these factors, we sought to identify potential areas for improvement in providing more individualized, inclusive, sensitive, and patient-centered care for all individuals, regardless of their sexual orientation or gender identity. Moreover, we aimed to shed light on disparities in healthcare experiences between heterosexual and LGBTQ+ patients, contributing valuable insights for obstetricians–gynecologists and other healthcare practitioners to enhance the quality of care provided to diverse populations.

## 2. Materials and Methods

This research was conducted in three stages, starting on 2 November 2023 and continuing until 3 January 2024, and the final results were based on the data collected from an online anonymous survey conducted in the Lithuanian language. Study participants were individuals identifying themselves as a part of the LGBTQ+ community and heterosexual individuals whose biological sex is female. In the first stage, prior to designing the survey, interviews were conducted with members of the Lithuanian LGBTQ+ community whose biological sex was female, and their responses were used to shape the questionnaire. Once the questionnaire was prepared, respondents were invited to take part in the survey, which was made publicly available on social media for the targeted groups, with an explanation of its importance for the evaluation and further development of OBGYN care in Lithuania for both the LGBTQ+ community and heterosexual women. Recruitment was conducted exclusively via social media platforms such as “Facebook” and “Instagram”. While this allowed for targeted outreach and broader participation from diverse communities, it may have introduced a selection bias toward younger, more internet-literate individuals who are active on these platforms. In the beginning, LGBTQ+ community respondents were invited to participate, and the top age limit for the heterosexual group was chosen based on the age of the oldest LGBTQ+ respondent in order to make the two comparison groups as similar as possible.

The online questionnaire was divided into four parts. In the first part, the demographic data and sex life characteristics of all respondents were collected. In the second part, data were collected on the respondents’ gender, social identity, belonging to the LGBTQ+ community, and preferred pronouns. In Section 3, from respondents who have visited an OBGYN, information was collected about the experience and other features of their visits to an OBGYN, the frequency of visits, and reasons, and from those who have never visited an OBGYN, information was collected about the reasons for not visiting. In Section 4, information was collected about respondents’ obstetrical and gynecological anamnesis and general health status, such as non-gynecological/chronic illnesses and use of prescription drugs. An English translation of the full questionnaire is available in Appendix A.

Statistical analysis of the data was performed in the MS Excel 2021 (Microsoft Corporation, Redmond, WA, USA) and RStudio Desktop programs (Posit PBC, Boston, MA, USA). The chi-square test of independence was used for the analysis of qualitative variables. The results of the analysis were described in terms of frequencies and relative frequencies (%). The results were considered reliable when *p* ≤ 0.05.

## 3. Results

### 3.1. Characteristics

Two hundred and fifty-six respondents aged 18 to 45, whose biological gender is female, took part in the survey. Of the 256 respondents, 127 identified themselves as LGBTQ+ and 129 were heterosexual. The median age of LGBTQ+ respondents in the final sample was 23 [20.00; 26.00], and the median age of the heterosexual group was 26 [21.00; 23.00].

All heterosexual respondents answered the question about pronouns with which they would like to be addressed and chose the option “she/her”—128 (99.2%) respondents. In the group of respondents who identify themselves as LGBTQ+, the most popular pronoun by which respondents would like to be addressed was also “she/her”, which was chosen by 115 (90.6%) of respondents (Table 1).

After a more detailed analysis of the gender and sexual identities of respondents from the LGBTQ+ community, it was found that the majority of LGBTQ+ respondents considered themselves bisexual 59 (46.5%) and homosexual 40 (31.5%) (Supplementary Table S1). A total of 59 (46.5%) LGBTQ+ respondents are open about their sexual and gender identity in society. Comparing the sexual identities of publicly declaring and non-declaring respondents, it was observed that LGBTQ+ respondents who self-identified as homosexual declared their sexual identity publicly more often: 25 (42.4%) vs. 15 (22%), accordingly (*p* = 0.0142). This trend and its statistical significance also remained when respondents’ gender and sexual identity were considered: 23 (39%) respondents of the LGBTQ+ group, whose gender and sexual identity coincided, publicly declared their sexual identity, and 15 (22%) did not (*p* = 0.0378). Nevertheless, among LGBTQ+ respondents who identify as bisexual, statistically significantly more respondents chose not to publicly declare their sexual identity: 21 (35.6%) vs. 38 (55.9%), accordingly (*p* = 0.0222). However, comparing only responses of those LGBTQ+ bisexual respondents whose gender and sexual identities coincided, the difference between the groups was statistically insignificant, 21 (35.6%) vs. 32 (47.1%), with *p* = 0.1913. See Supplemental Table S1.

### 3.2. Sexual Habits

A total of 167 (65.2% of all questionnaire respondents) respondents currently have a regular partner, of which 93 (72.1%) are heterosexual respondents and 74 (58.3%) are LGBTQ+ respondents. A statistically significantly higher number of heterosexual persons currently have a regular partner compared to representatives of the LGBTQ+ community (*p* = 0.0202).

However, there was no statistically significant difference between LGBTQ+ and heterosexual groups in the proportion of persons having regular sexual relations (*p* = 0.1023): 84 (65.1%) heterosexual persons and 70 (55.1%) persons from the LGBTQ+ group had regular sexual relations.

The majority of respondents from the LGBTQ+ group only had sexual relations with women at the time of filling out the questionnaire (40.9%). Additionally, 16.5% of respondents from the LGBTQ+ group had sexual relations only with men at the time of filling out the questionnaire, and 9.5% with both sexes. At the time of filling out the survey, 57 (44.9%) respondents belonging to the LGBTQ+ group did not have regular sex. In the group of heterosexual persons, at the time of filling out the survey, 45 (34.8%) respondents had no sexual relations at all, and the rest had sexual relations only with men.

In total, 91 (71.7%) respondents from the LGBTQ+ group and 15 (11.6%) respondents from the heterosexual group have had sexual relations with women. This difference was statistically significant, *p* < 0.0001.

According to the survey, LGBTQ+ respondents use sex toys slightly more often, but this difference was not statistically significant: 67 (51.9%) heterosexual persons vs. 79 (62.2%) LGBTQ+ persons (*p* = 0.0971). A statistically significantly higher proportion of respondents from the LGBTQ+ community share their sex toys with their partner compared to heterosexual respondents—47 (31.5%) LGBTQ+ respondents and 19 (28.4%) heterosexual respondents (*p* = 0.0002).

According to the questionnaire, barrier contraceptives were used statistically significantly more frequently by heterosexual respondents compared to LGBTQ+ group respondents (*p* = 0.0097): barrier contraceptives were used by almost half of heterosexual persons (47.3%) and about a third of LGBTQ+ persons—31.5%. All the people who filled out the survey and used barrier contraceptives chose to regularly use only male condoms out of all the available barrier contraceptives.

The incidence of sexually transmitted diseases was not statistically different between LGBTQ+ and heterosexual respondents: 12 (9.3%) heterosexual individuals and 14 (11%) LGBTQ+ individuals have ever had an STI.

### 3.3. Female-Controlled Barrier Contraception Methods

Female-controlled barrier contraceptive methods include female condoms, diaphragms, sponges, and cervical caps [7]. In total, 202 respondents knew that female barrier contraceptives existed. One hundred (78.7%) respondents were aware of female barrier contraceptives among respondents who identify themselves as LGBTQ+, compared to 102 (79%) respondents among heterosexual patients. Statistically significantly more LGBTQ+ respondents answered that they know how to use them compared to the group of heterosexual respondents: 59 (59%) vs. 43 (42.1%), accordingly (*p* = 0.0167). Interestingly, persons of the heterosexual group said statistically significantly more often that they know where female barrier contraceptives can be purchased in Lithuania (36.3%), compared to persons of the LGBTQ+ group (22%) (*p* = 0.0257).

However, only 8 (6.2%) heterosexual respondents and 13 (10.2%) LGBTQ+ respondents have ever used female barrier protection at least once in their life—21 respondents out of 256 in total. Of those, only 11 respondents would use this type of contraception if it was more easily accessible in Lithuania—10 (76.9%) LGBTQ+ and 1 (12.5%) heterosexual. Of the respondents who would choose to use female barrier contraception, five (50%) respondents currently have sexual relations with a woman, two (20%) with men, one (10%) with both sexes, and two (20%) currently do not have sexual relations. Among these respondents, the main reasons for hesitation to use this method were discomfort and price.

Among all women, the most common reasons for not using female barrier contraception were that this barrier contraception method was considered not comfortable (mentioned by 49 (50%) heterosexual respondents and 32 (41%) LGBTQ+ respondents) and not necessary for them (20 (20.4%) heterosexual respondents and 21 (26.9%) LGBTQ+ respondents). The differences did not differ significantly between LGBTQ+ and heterosexual groups. After separately analyzing the reasons of the respondents who have tried using female barrier contraceptives and those who have not regarding why they would not choose to use female barrier contraceptives if their availability were better in the Lithuanian market, it became clear that respondents who tried female barrier contraceptives would mostly not choose them because of discomfort (3 (30%)) and high price (3 (30)%), while those who have never tried to use female contraception due to perceived discomfort numbered 78 (47%) (Table 2 and Table 3).

### 3.4. Birth Pill Usage

When comparing the frequency of use of combined contraceptive pills (CCPs) between the LGBTQ+ group and heterosexual respondents, no statistically significant differences were found, but a tendency was observed that heterosexual respondents chose to use them more often: they took or used to take compound contraceptive pills at some point in their life at the time of filling out the questionnaire, including 34 (26.4%) heterosexual respondents and 21 (16.5%) LGBTQ+ respondents (*p* = 0.0558).

The majority of heterosexual survey participants noted that they used CCPs to prevent pregnancy (15 (75%)) and slightly less to regulate the menstrual cycle (12 (60%)). In the LGBTQ+ group, the most common reason for using CCPs was menstrual cycle disorders—for this reason, nine (69.2%) survey participants of the LGBTQ+ group used CCPs, while only four (30.8%) respondents belonging to the LGBTQ+ group used it to prevent pregnancy (Supplementary Table S2).

According to the questionnaire, more than half of the respondents using CCPs said that their libido decreased when taking CCPs—a total of 29 (52.7%). Of the remaining respondents, 22 (40%) respondents answered that their libido did not change when taking CCPs, and 4 (7.3%) that their libido increased. There were no statistically significant differences in the effect of CCPs on libido between the LGBTQ+ group and the group of heterosexual respondents (Supplementary Table S3).

### 3.5. Visits to OBGYN

Of all the respondents who took part in the survey, a total of 225 respondents visited an obstetrician–gynecologist at least once in their life—114 (88.4%) persons of the heterosexual group and 111 (87.4%) persons of the LGBTQ+ group. The age of the first visit to an OBGYN for both groups of respondents did not differ statistically significantly—the respondents visited an OBGYN for the first time, on average, around the age of 18. Among the respondents of the LGBTQ+ group who had a first visit to a doctor, the average age when they first visited a gynecologist was 18.05, and that of heterosexual respondents was 18.16. A total of 16 (12.65) LGBTQ+ and 15 (11.6%) heterosexual respondents never visited an OBGYN. After analyzing the reasons for the first visit to an OBGYN, it was found that 42 (36.8%) heterosexual persons and 44 (39.6%) persons from the LGBTQ+ group visited an OBGYN for the first time as a preventive measure—the rest of the respondents went to the gynecologist for the first time already having complaints (Supplementary Table S4).

Two-thirds of respondents who identify themselves as LGBTQ+ (71 (67%)) already considered themselves part of the LGBTQ+ community when they first visited an OGBYN. However, out of 127 members of the LGBTQ+ community who filled out the questionnaire, only 20 (18%) of the individuals’ doctors knew that they belonged to the LGBTQ+ community, although 54 (48.6%) LGBTQ+ community respondents who visited an OBGYN publicly declared their gender and sexual identity in society. Thus, more than half of those who publicly declare their gender and sexual identity chose not to disclose it to their OBGYN for some reason.

At the time of filling out the survey, a total of 86 (77.5%) LGBTQ+ and 104 (91.2%) heterosexual respondents regularly visit an OBGYN. The remaining respondents did not regularly visit an OBGYN at the time of filling out the survey. Thus, statistically significantly more respondents from the LGBTQ+ group (25 (22.5%)) at the time of filling out the questionnaire did not regularly see a doctor, compared to heterosexual respondents (10 (8.8%)), with *p* = 0.0044.

Among the patients who visited the OBGYN regularly, the majority of respondents from the LGBTQ+ group visited the OBGYN once a year—43 (37.7%)—and the majority of heterosexual respondents visited one once every 2–3 years—28 (24.6%). No statistically significant differences were observed between LGBTQ+ and heterosexual respondents in the frequency of visits to the gynecologist (Table 4).

After analyzing the reasons for which respondents visited an OBGYN in the last two years, the most common reasons for respondents from the LGBTQ+ group and heterosexual respondents coincided. In the last two years, the respondents most often went to the OBGYN for a preventive check-up; due to menstrual cycle disorders; due to organic pathology, e.g., ovarian cysts, endometrial and cervical polyps, and fibroids; and a little less often due to vulva/vaginal infections and candidiasis. There were no statistically significant differences between reasons for visiting an OBGYN between LGBTQ+ and heterosexual patients (Table 5). Moreover, at the moment of filling in the survey, 51 (40.2%) of LGBTQ+ respondents and 39 (30.2%) of heterosexual respondents claimed to have any gynecological complaints, and these rates difference were not significantly different between heterosexual and LGBTQ+ respondents (*p* = 0.116).

Of the 20 respondents whose doctors knew they were LGBTQ+, only a quarter (25%) said their OBGYNs asked them additional questions that they would not have asked otherwise. Interestingly, respondents who were not able to openly disclose their sexual identity to the OBGYN have mentioned that if they were able to do so, they would be eager to ask their OBGYNs about sexually transmitted diseases, possibilities to prevent these diseases while having sexual relations with the same sex, and the possibilities of reproduction while being in a same-sex relationship.

Alarmingly, almost a third (30%) of respondents from the LGBTQ+ community whose OBGYN learned about their gender and/or sexual orientation during their visit said that after learning this information about the patient, the doctor’s attitude towards the patient changed, i.e., they started treating them differently. What is more, six (5.4%) respondents belonging to the LGBTQ+ community say that the frequency of their visits to the OBGYN changed after they started to consider themselves part of the LGBTQ+ community.

After a more detailed evaluation of situations in the OBGYN office that LGBTQ+ women have to face and that can cause them negative emotions, we found that as many as 101 (91%) LGBTQ+ respondents faced a situation that could cause discomfort and that the OBGYN never asked about their sexuality and/or gender identity. Of the 20 respondents who told their OBGYN about their sexual and/or gender identity, only 5 (25%) were asked this question by their OBGYN, while others had to bring the topic up themselves. Also, as many as 110 (99%) of LGBTQ+ respondents in the survey indicated that their OBGYNs had never asked what pronouns they should be addressed by. Notably, not one of the LGBTQ+ respondents whose OBGYNs were informed about their patients’ sexual and/or gender identity was asked by their doctor about what pronouns they should refer by. In the group of heterosexual individuals, an even higher proportion of women said that the OBGYN never asked about their sexual and gender identity (97.4%) and never asked what pronouns they should be addressed by (97.4%). Even more, 93 (83.8%) of LGBTQ+ respondents have been asked by an OBGYN about sexual relations with a man without first asking about their sexual and/or gender identity. Out of 20 respondents whose OBGYNs knew they belonged to the LGBTQ+ community, as many as 18 (90%) have encountered this situation. Ninety-two (80.7%) heterosexual respondents also faced this situation. Only one (0.87%) of the respondents belonging to the heterosexual group and two (1.8%) of the respondents belonging to the LGBTQ+ group have been actively asked by their OBGYN about sexual relations with a woman.

As many as 76 (33.8%) of the survey participants have at least once in their lives been directly or indirectly urged by an OBGYN that they should have children at least once in their lives, being of the female biological sex. Respondents from the LGBTQ+ group have heard this doctor’s opinion statistically significantly more often compared to heterosexual respondents: 45 (40.5%) vs. 31 (27.2%), accordingly (*p* = 0.0343). Of respondents whose OBGYNs knew their patients were LGBTQ+, six (30%) have heard this statement.

Although nine (8.1%) persons managed to find an OBGYN in a public medical institution with whom they could openly talk about their sexual orientation and gender identity, and eight (7.2%) respondents found such an OBGYN in a private medical institution, only two (1.8%) respondents belonging to the LGBTQ+ group participants report asking their own OBGYN questions about same-sex intercourse. In addition, 35 (31.5%) respondents from the LGBTQ+ group and 26 (22.8%) heterosexual respondents said that in their environment, they have to hear feedback about the inappropriate attitude of OBGYNs towards patients and/or their treatment. A total of 13 (11.7%) of all survey respondents have heard about the inappropriate behavior/attitude of an OBGYN when an OBGYN learns that their patient belongs to the LGBTQ+ community.

### 3.6. Obstetrical–Gynecological Anamnesis and Reproductive Plans

At the time of filling out the survey, 39 (30.2%) heterosexual respondents and 51 (40.2%) participants belonging to the LGBTQ+ group claimed to have gynecological complaints, and the difference between these rates was not statistically significant.

A statistically significantly higher number of heterosexual persons who filled out the survey had been pregnant at least once in their life, compared to LGBTQ+ respondents: 41 (31.8%) vs. 11 (8.7%), accordingly (*p* = 0.0001). Of those who became pregnant, 32 (78%) and 7 (63.6%) chose to give birth in the heterosexual and LGBTQ+ groups (*p* = 0.3270). Among the respondents who were pregnant at least once in their life, medical or instrumental termination of pregnancy was performed at least one time for five (45.5%) LGBTQ+ group respondents and nine (22%) heterosexual respondents, accordingly (*p* = 0.1186).

According to the survey, a statistically significantly higher number of heterosexual respondents would like to have biological children in the future compared to the survey participants of the LGBTQ+ group: 73 (56.6%) vs. 43 (33.9%), accordingly (*p* = 0.0003). Also, statistically significantly more heterosexual respondents compared to the LGBTQ+ group already have their own biological children: 25 (19.4%) heterosexual respondents and 3 (2.4%) representatives of the LGBTQ+ group, accordingly (*p* = 0.0001).

### 3.7. Other Factors That May Affect Gynecological and Obstetric Pathologies and Their Outcomes

According to the collected data, 59 (46.5%) individuals in the LGBTQ+ group and 44 (34.1%) in the group of heterosexual respondents smoked. There were 12.4% more smoking individuals in the LGBTQ+ group than in the group of heterosexual respondents—the difference between the groups was statistically significant (*p* = 0.0373). Two heterosexual and three LGBTQ+ respondents did not answer the question about smoking (Table 6).

The majority of smoking respondents, both those who identify themselves as LGBTQ+ and heterosexuals, smoked 1–2 cigarettes per day at the time of filling out the questionnaire: 19 (32.2%) and 9 (20.5%), respectively. Respondents of the LGBTQ+ group were statistically significantly more likely to smoke five or fewer cigarettes per day compared to the group of heterosexual respondents (*p* = 0.0027). Heterosexual respondents were statistically significantly more likely to smoke electronic cigarettes (*p* = 0.0172) (Table 7).

The number of alcohol drinkers in heterosexual and LGBTQ+ respondents was very similar: 77.5% and 78.7%, respectively. Twenty-nine heterosexual individuals and twenty-five LGBTQ+ individuals did not consume alcohol. There were no statistically significant differences in alcohol consumption patterns between heterosexual and LGBTQ+ drinkers (Supplementary Tables S5 and S6).

A statistically significantly higher proportion of LGBTQ+ respondents answered that they had used psychoactive substances compared to the group of heterosexual respondents: 26 (43.3%) and 9 (7%), accordingly (*p* = 0.0013). The majority of heterosexual and LGBTQ+ respondents who use psychoactive substances answered that, on average, they use them once a year—seven (27%) and three (37.5%), respectively. In both the heterosexual and LGBTQ+ community respondent groups, there were individuals who used psychoactive substances with different frequency—from an average of one time per year to more than one time per week. Respondents of the LGBTQ+ group chose all frequency of use options slightly more often compared to heterosexuals, but due to the small sample of respondents using psychoactive substances, no statistically significant differences between the groups were observed (Table 8 and Table S7).

A total of 102 (39.8%) respondents engaged in regular physical activity. LGBTQ+ individuals engaged in regular sports 6.9% more often than heterosexual respondents—55 (43.3%) members of the LGBTQ+ community and 47 (36.4%) heterosexual respondents—but this difference was not statistically significant. The majority of both heterosexual and LGBTQ+ respondents engaged in regular sports 2–3 times a week: 27 (57.4%) and 28 (50.9%), respectively (Supplementary Tables S5 and S6). Also, according to the questionnaire data, none of the survey participants used anabolic steroids.

### 3.8. Non-Gynecological Diseases Possibly Affecting Gynecological and Obstetrical Care

In the questionnaire, respondents were asked to name all other non-gynecological diseases that they were diagnosed with as well as about certain medications that they were taking at the moment of filling in the questionnaire. Comparing the frequency of depression between LGBTQ+ and heterosexual respondents, it was found that twice as many LGBTQ+ respondents had depression when filling out the questionnaire compared to heterosexuals, and this difference is statistically significant—35 (27.6%) vs. 16 (12.4%), accordingly (*p* = 0.0024). According to the questionnaire data, four (3.1%) respondents from the LGBTQ+ group and two (1.6%) from the heterosexual group had other psychiatric disorders.

Thyroid dysfunction was reported by four (3.2%) LGBTQ+ and seven (5.4%) heterosexual respondents. The frequency of individuals claiming to have thyroid disease was not statistically significantly different between LGBTQ+ and heterosexual groups (*p* = 0.3691). A small number of respondents also suffered from such chronic diseases as diabetes, arthritis, asthma, migraine, chronic gastritis, and skin diseases, but these cases were isolated and not significantly more frequent than one of the groups.

A total of 80 (63%) respondents of the LGBTQ+ group and 68 (52.7%) respondents of the heterosexual group have had a urinary tract infection at least once in their life—this difference was not statistically significant (*p* = 0.0568) (Supplementary Table S7).

According to the questionnaire answers, the majority of all respondents who have had urinary tract infections in their life have them less than once a year: 64 (80%) respondents in the LGBTQ+ group and 44 (64.7%) respondents in the heterosexual group. Over a period of a year and more often, heterosexual persons suffered from urinary tract infections more often than respondents from the LGBTQ+ community: 20 (29.5%) vs. 13 (16.4%), respectively. However, the number of respondents was too low for the difference to be statistically significant (*p* = 0.0552) (Table 9).

When the weight, height, and BMI of heterosexual and LGBTQ+ patients were evaluated, no statistically significant differences were found between the BMI categories of the groups (Supplementary Table S8).

## 4. Discussion

According to the “LGBT+ Pride 2021 Global Survey”, conducted by Ipsos between April 23rd and May 7th, 2021, only 80% of respondents globally identify themselves as heterosexual. The investigation, conducted in 27 different counties and surveying 19,069 people aged 16–74, revealed that, on average, the other 20% of the world‘s population is distributed accordingly: gay, lesbian, or homosexual—3%; bisexual—4%; pansexual or omnisexual—1%; asexual—1%; “other”—1%; do not know or will not say—1% [8].

In the “LGBT+ Pride 2023” global survey, conducted in 30 different counties and surveying 22,514 people aged 16–74, some interesting patterns were also observed regarding generational differences, proving that society is becoming more diverse with every decade, demanding more open-minded healthcare services. For example, respondents born between 1997 and 2012 were found to identify themselves as bisexuals (9%) much more frequently in comparison with respondents born between 1981 and 1996 (4%), respondents born between 1965 and 1980 (2%), and respondents born between 1946 and 1964 (2%). Also, younger individuals were noted to identify themselves as homosexual, pansexual/omnisexual, and asexual more often than previous generations [9].

When the results regarding the rates of LGBTQ+ community members in the general society are compared between different countries, the results are often also ambiguous. For example, in the previously mentioned study by Ipsos of 2021, 39% of respondents in Malaysia, 33% in Turkey, 24% in India, 19% in Russia, and 15% in Mexico were unable or unwilling to define their sexual orientation. However, this may be due to a variety of reasons, from cultural differences to various levels of tolerance and discrimination in certain countries; therefore, these frequencies are not enough to determine whether the respondents are actually afraid to disclose their identities or unable to choose between the vast variety of possible options available and “acceptable” in their culture. Several assumptions regarding the unwillingness to disclose their identities could be drawn from the data regarding respondents’ identification: in Brazil, Spain, Australia, Canada, and the Netherlands, about 5% of respondents identified themselves as lesbian/gay/homosexual, while in Hungary, Peru, Italy, Poland, Japan, China, and South Korea, only 1% did so, and in Russia, less than 1% did so [8].

This study is the first study carried out in Lithuania analyzing the characteristics of LGBTQ+ individuals, whose biological sex is female, and their healthcare in relation to the field of obstetrics and gynecology. As Lithuania is a relatively small country with a population of only 2,854,099 in 2023, the size of the LGBTQ+ community in Lithuania is also relatively small [10]. However, due to widespread discrimination against the members of the LGBTQ+ community, openness about sexual and gender identity is still not common in Lithuania, and the majority of LGBTQ+ individuals tend to hide their true selves [11,12]. According to a study by the European Union Agency for Fundamental Rights (FRA) surveying 139,799 individuals from the LGBTQ+ community in 2019, 60% of the Lithuanian LGBTQ+ population was never or rarely open about their sexual and gender identity [13]. Additionally, Lithuania had the highest proportion of LGBTQ+ community members in the EU who felt discriminated against at work (32%). In comparison, in Denmark and Sweden, the proportion was 5% and 6%, and in Finland and the Netherlands, it was 6% [13]. Lithuanian LGBTQ+ individuals were also found to have one of the highest rates of not reporting physical or sexual attacks due to the fear of homophobic and/or transphobic reactions from the police [13]. Moreover, more than 40% of LGBTQ+ respondents from Lithuania, together with individuals from Poland, Bulgaria, Romania, Croatia, Hungary, and France, indicated that they often or always avoid certain places or locations due to fear of being assaulted, threatened, or harassed [13]. Taking all of this into consideration, it is quite understandable as to why the actual number of individuals belonging to the LGBTQ+ community is still unknown in our country. Therefore, we believe that the number of LGBTQ+ respondents whom this survey has reached and who agreed to share their experiences anonymously is satisfactory to represent the situation of the LGBTQ+ community in Lithuania quite accurately.

There are many widely spread beliefs regarding the individuals of the LGBTQ+ community: their specific lifestyle habits and health issues [14]. Some of these beliefs are based on actual studies, while others may have formed without any specific proof. Moreover, LGBTQ+ individuals suffer from discrimination, lack of empathy, and premature assumptions regarding their lifestyle more frequently than heterosexuals [15]. Therefore, some LGBTQ+ individuals choose to keep their gender/sexual identity hidden in order to avoid being treated differently [5]. In healthcare, this can cause significant issues as it may prevent healthcare professionals from fully understanding the specific situations or needs of their patients. Moreover, healthcare professionals may unknowingly ask unsuitable questions or make assumptions that are offensive to LGBTQ+ individuals, leading to their further dissociation from their sexual/gender identity.

In our study, bisexual individuals made up the biggest portion of LGBTQ+ respondents, with homosexual individuals being the second biggest group. Interestingly, bisexual respondents tended to not disclose their sexual and gender identity statistically more frequently than homosexual individuals. This result coincides with the research published in 2016 by the Journal of Child and Family Studies [16,17,18]. Understanding this tendency, an obstetrician/gynecologist should be aware of the possibility of already having bisexual patients in his/her practice without knowing it. Additionally, although the majority of both groups chose to be addressed as “she/her”, it is important to note that there were about 10% of LGBTQ+ respondents who chose other pronouns. Therefore, in order to not offend anyone, an OBGYN should avoid making assumptions regarding patients’ gender and sexual identity and should always inquire first before carrying on with the consultation.

One of the important facts for an obstetrician/gynecologist to be aware of regarding individuals who identify as part of the LGBTQ+ community is their increased tendency towards addictions. Several studies claim that LGBTQ+ individuals tend to be more frequently addicted to alcohol, psychoactive substances, and smoking [19,20,21]. Our study found statistically significant differences between heterosexual and LGBTQ+ individuals only in regard to the use of psychoactive substances and smoking: LGBTQ+ respondents tended to adopt these lifestyle habits more frequently than heterosexual respondents.

However, no differences between groups were observed regarding alcohol consumption. It is important that an OBGYN is aware of the increased risk of possible addictions among the members of the LGBTQ+ community in order to assess the risk and suspect the possible causes of certain health issues, such as congenital disabilities caused by the consumption of psychoactive substances or intrauterine growth restriction that may be caused by smoking during pregnancy [22,23]. Furthermore, smoking is known to increase the risk of thrombotic events for women taking birth control pills [24]. In our survey, only five LGBTQ+ individuals took birth control pills while smoking and their age was between 20 and 24 years, so the risk for thrombotic events was not as high as it would be for older smoking individuals. Nevertheless, it is important that these individuals are informed about the increased risks associated with smoking. Moreover, studies suggest that individuals identifying as part of the LGBTQ+ community have an increased risk of suffering from depression or other mental disorders. This increased risk can be a result of experiencing such stressors as discrimination, bullying, etc. [25,26]. Our study determined that a statistically significantly higher number of LGBTQ+ respondents had depression compared to heterosexual respondents. It is important that an OBGYN is aware of the increased risk of depression in their patients, as depression may affect patients’ compliance with treatment. Knowing this, an OBGYN could choose a different management strategy that is more suitable for the individual.

As women are at higher risk of developing depression in specific reproductive periods of vulnerability such as adolescence, pregnancy, postpartum, and the menopausal transition, OBGYN can be an important link in the healthcare system, helping to suspect the development of depression in the primary stages and referring the patient to other specialists in time [27]. Moreover, although it was not reflected in our study, it is important to mention that members of the LGBTQ+ community are at a higher risk of suffering not only from depression but also from other mood or anxiety disorders [28].

Recent research has also shown that members of the LGBTQ+ community may be at increased risk not only for mental health disorders but also for chronic illnesses and cancer [29]. In our study, there were no statistical differences in the prevalence of chronic illnesses between the two groups; however, such results could be influenced by an insufficient number of respondents for these subtle tendencies to be detected.

What is more, studies show that members of the LGBTQ+ community lack sex education, for example, information on how to protect themselves during oral sex or how sexually transmitted infections (STIs) can be transmitted [30]. An Australian study published in 2022 reported that certain sexually transmitted diseases were more prevalent depending on the gender of a woman’s sexual partners: women who have had sex with a woman were more likely to be infected with bacterial vaginosis, whereas women who have had sex with a man were more likely to be infected with chlamydia. However, the study suggests that this problem is equally relevant for both heterosexual and LGBTQ+ individuals [31]. No differences were observed in the prevalence of STIs between the two groups. This finding indicates that both groups of respondents were exposed to sexually transmitted infections, making this problem equally important for both LGBTQ+ and heterosexual individuals. Thus, it is important for OBGYNs to know that STIs can occur not only in patients who have sex with men but also with women [32]. In our study, no statistically significant difference was found between LGBTQ+ and heterosexual respondents regarding the rates of STIs; therefore, our study also supports the idea that it is as important for LGBTQ+ individuals to practice safe sex as it is for heterosexual couples. Our study also determined that a higher number of LGBTQ+ respondents had ever suffered from a urinary tract infection in their lifetime, and a higher number of them had had a urinary tract infection once a year or more frequently, in comparison to heterosexual individuals. As this infection is more common among women in general and is also often associated with sexual activity, lack of hygiene, and excessive use of intimate hygiene products, which destroys the natural microbiome of the vagina, there is evidence that sexual minority women are at higher risk for urinary tract infections [33,34]. It is important for an OBGYN to have this fact in mind when consulting patients in order to offer them suitable prophylactic strategies to avoid the recurrence of this pathology in the future.

In our study, a significantly higher number of heterosexual respondents had a regular sex partner in comparison with LGBTQ+ respondents. However, the number of respondents having regular sexual intercourse did not differ significantly between LGBTQ+ and heterosexual groups. Therefore, a hypothesis can be raised that LGBTQ+ individuals who do not have a regular partner tend to have more sexual relations with different partners, increasing their risk of contracting an STI. Furthermore, studies have established a tendency for more frequent use of sex toys among LGBTQ+ women in comparison with heterosexual women. Our study has also observed this. Research conducted in 2010 and 2011 suggests that women in the LGBTQ+ community are more likely to use sex toys during sexual intercourse in comparison with heterosexual respondents [35,36]. More frequent use of sex toys during intercourse may lead to more frequent sharing of sex toys between partners. Our study has also verified this proposition as LGBTQ+ individuals appeared to share their sex toys with their partners statistically significantly more frequently.

Our study has also established that only one-third of all LGBTQ+ respondents used barrier contraceptives during sex, whereas almost half of all heterosexual individuals did. This tendency was also confirmed by previous studies. For example, 2018 research published in the *Journal of Adolescent Health* in 2018 claimed that women who have sex with other women of the same sex rarely use female barrier contraceptives (e.g., dental dams) due to a lack of knowledge about safe sex methods available for having sex with a woman [37,38]. Although the only regularly used barrier contraceptive reported in the survey was male condoms, the majority of both LGBTQ+ and heterosexual individuals claimed to know about the existence of female-controlled barrier contraceptives. Therefore, a lack of knowledge about the variety of barrier contraceptives cannot be considered the reason for not using them. Moreover, LGBTQ+ individuals were statistically significantly more likely than heterosexual respondents to know how to use female-controlled barrier contraceptives in theory. This could be explained by a higher demand for this kind of contraceptive for sexual behavior specific to some LGBTQ+ individuals, where there are fewer contraceptive possibilities in general. However, only a small number of the members of the LGBTQ+ community indicated ever trying female-controlled barrier contraceptives in the past. This may be due to the fact that it is difficult, if not impossible, to purchase female-controlled barrier contraceptives in the Lithuanian market. When all respondents who have ever tried this type of contraception were asked if they would choose to use female barrier contraception if it were more easily accessible in Lithuania, the majority of LGBTQ+ respondents replied positively, while there was only one positive answer from the heterosexual respondent group. In the group of respondents who have never tried female barrier contraception, only one-eighth of all survey respondents expressed willingness to try it if it were more accessible. When the reasons behind this hesitance were analyzed, the majority of participants stated that they would not use female barrier contraception due to its inconveniency or perceived implied inconveniency. Among respondents who have tried this method, the high cost was identified as just as important a factor as convenience. The low usage may also reflect cultural stigma around female-initiated contraception and the lack of visibility of such methods in mainstream sexual education. Additionally, limited availability in Lithuanian pharmacies and healthcare systems further restricts access. In research published in 2018, it was also stated that LGBTQ+ participants reported not using female barrier contraception during same-sex intercourse due to discomfort. Other cited reasons were decreased pleasure and lack of knowledge about safe sex and barrier contraception [37]. A recent systematic review found that limited availability of female barrier methods, high costs, and inadequate healthcare infrastructure restrict access to these contraceptive options [39].

Due to such practices as sharing sex toys with a partner, not washing them between uses, and not using barrier contraceptive methods, LGBTQ+ individuals are as likely to contract STIs, HPV, bacterial vaginosis, and vaginal candidiasis as heterosexual women [40]. Furthermore, as LGBTQ+ individuals tend to avoid visiting an OBGYN, even for routine screenings, untreated STIs could also lead to more serious health issues in the future, such as infertility or cervical cancer [41,42,43].

Professional obstetrical/gynecological care is an important factor in the well-being of every person whose biological sex is women. Irrespective of sexual and gender identity, a patient must always have a right to solve their health problems discretely and with the help of a professional healthcare specialist [44]. Understanding that both LGBTQ+ and heterosexual women are at considerable risk of contracting an STI, the OBGYN should ensure to provide suitable and reliable information regarding protective measures and safe sex practices for each specific case individually. And for that, the OBGYN needs to create a safe environment where every patient can speak openly about their identity and sexual behavior. When knowing about a patient’s sexual and gender identity, an OBGYN cannot only better understand the needs of a specific patient but also more easily diagnose certain conditions that LGBTQ+ individuals are more prone to, such as bacterial vaginosis [45]. Unfortunately, according to recent studies, most LGBTQ+ members fear that the quality of care will be negatively affected if they disclose their sexuality or gender identity [4,5]. Nevertheless, according to a European Union Agency for Fundamental Rights (FRA) study surveying 139,799 individuals from the LGBTQ+ community, openness about assigning themselves to this community affects individuals’ life satisfaction rates [13]. Therefore, openness about their sexuality and gender identity could not only directly benefit the healthcare of members of LGBTQ+ community but would also improve their general life satisfaction, leading to fewer depressive disorders in the long term.

Although the number of heterosexual and LGBTQ+ respondents who had ever visited an OBGYN and the age of the first visit did not differ significantly between groups, it is important to note that the majority of LGBTQ+ respondents did not disclose their sexual and gender identity to the physician during their OBGYN appointments. This occurred despite two-thirds of them already identifying as part of the LGBTQ+ community during their first visit to an OBGYN. This indicates that LGBTQ+ respondents did not feel comfortable to openly discuss their identity. Furthermore, our study revealed that the majority of all respondents were never asked by their OBGYN about their gender and sexual identity or the pronouns they should be addressed by and were assumed to be having sexual relations with a man without further inquiry. On top of other possible tactless remarks by the OBGYN, such as suggesting that individuals of female biological sex should have children, this may contribute to discomfort during OBGYN appointments for LGBTQ+ individuals and could be one of the factors influencing their decision to attend future visits less frequently. Interestingly, the latter proposition was statistically significantly more frequently reported by LGBTQ+ respondents, raising the question of whether OBGYNs feel the urge to mention this more often to this group of individuals or whether LGBTQ+ individuals tend to be more sensitive to comments regarding this issue.

The majority of individuals from both groups of our survey respondents visited an OBGYN for the first time due to certain health complaints, demonstrating an equal demand for appropriate and accessible OBGYN care among both heterosexual and LGBTQ+ individuals. However, over the past two years, more than half of heterosexual respondents visited an OBGYN prophylactically, while in the LGBTQ+ group, the number of prophylactic visits was 10% lower. It is unclear whether this difference is due to the general reluctance of LGBTQ+ individuals to visit an OBGYN without an urgent need or as the result of a higher incidence of gynecological health issues encountered by heterosexual individuals during the past two years. Other reasons for visiting an OBGYN in the last 2 years did not differ significantly between LGBTQ+ and heterosexual respondents, with menstrual cycle disorders, ovarian cysts, endometrial and cervical polyps, fibroids, and vulvovaginal infections being the most common. Additionally, at the time of filling in the survey, a higher number of LGBTQ+ respondents reported gynecological complaints compared to heterosexual respondents. Therefore, it is important to note that both LGBTQ+ and heterosexual individuals of female biological gender have experienced similar gynecological issues that require professional care. However, our study concluded that LGBTQ+ respondents visited an OBGYN statistically less frequently than heterosexual respondents. Moreover, 5.4% of respondents in the LGBTQ+ group indicated that their frequency of seeing an OBGYN changed after they began to identify as part of the LGBTQ+ community. It can be assumed that this happened due to a change in the attitude of the OBGYN or the fear of having unpleasant experiences after disclosing one’s sexual or gender identity.

Such lifestyle habits as more frequent smoking and the usage of psychoactive substances, as well as a tendency to avoid going to the OBGYN as frequently as it is necessary, may create an impression that LGBTQ+ individuals are not as concerned about their health as heterosexual individuals. However, contrary to such an assumption, our study observed a trend that LGBTQ+ respondents engaged in regular physical activity significantly more frequently than heterosexual respondents. This indicates that it would be inaccurate to claim that this community is generally unconcerned about their health. Moreover, the average age at the first visit to the gynecologist was almost the same for both groups, suggesting that LGBTQ+ individuals were not generally opposed to taking care of their gynecological health—rather, they may avoid regular OBGYN check-ups, possibly influenced by previous negative experiences.

What causes even more concern is that even healthcare professionals are not always prepared to admit patients from the LGBTQ+ community. A 2021 study from Illinois, USA, concluded that OBGYNs are insufficiently trained and prepared to provide appropriate healthcare for LGBTQ+ individuals. As many as half of the respondents stated that they do not feel equipped to provide adequate healthcare to lesbian and bisexual women, and more than 70% of the respondents feel unprepared to care for transgender people [46]. This tendency was also observed in our survey, as only five LGBTQ+ respondents (25% of those whose doctors knew about their sexual and gender identity) reported that their OBGYN asked them additional questions that they would not have asked otherwise. These data highlight an important issue: the majority of medical professionals in Lithuania are not equipped to meet the specific healthcare needs of LGBTQ+ patients. Additional education on this topic is needed. Moreover, of all 225 respondents, only 12 reported ever being actively asked about their sexual and gender identity by an OBGYN, and only 4 were asked what pronouns they should be addressed by.

The main limitation of our study is the relatively small LGBTQ+ respondent group size. However, considering the population size of Lithuania, the sensitivity of the subject, and the previously presented situation in which the LGBTQ+ community found itself in Lithuania at the time of the survey, we believe that the number of responses collected from LGBTQ+ individuals of the female sex is significant and representative. Another limitation of our study is the relatively young age of the respondents, which may not adequately represent the issues dealt with by the older generation of the LGBTQ+ community in Lithuania. Due to the young age of the study respondents, there may not be sufficient data to analyze the experiences and quality of obstetrical care for the LGBTQ+ community. Only 11 of the LGBTQ+ respondents in our study were ever pregnant, and only 7 chose to give birth. Additionally, recruiting participants through social media may have biased the sample toward younger and more digitally connected individuals. This may limit the generalizability of our findings to older or less internet-savvy populations. Therefore, further studies should be conducted, and additional measures should be taken in the future in order to reach older LGBTQ+ community members and uncover the specific issues they have encountered and continue to face in Lithuania in relation to the field of obstetrics and gynecology. While we attempted to match groups based on age to ensure comparability, we did not control for other potential confounding factors such as education level, urban versus rural residency, or socioeconomic status. These factors may have influenced healthcare access, health behaviors, and attitudes toward OBGYN visits and should be considered in future research. However, it is important to note that older individuals of the LGBTQ+ community in Lithuania may be private and less willing to share their personal experiences or even disclose their real gender or sexual identity due to having endured greater levels of discrimination in previous decades when the LGBTQ+ community was even more marginalized in Lithuania. While this study focused on quantitative analysis, future research should incorporate qualitative methods, such as interviews or open-ended surveys, to more deeply explore individual perspectives and healthcare experiences among LGBTQ+ patients.

## 5. Conclusions

This study is the first in Lithuania to analyze the characteristics of LGBTQ+ individuals whose biological sex is female in relation to the field of obstetrics and gynecology. Since OBGYN care for LGBTQ+ individuals remains an under-researched topic in Lithuania, the data collected in this study are key to understanding various aspects of the health and lifestyle characteristics of this community in Lithuania. In order to improve the education of medical professionals, potential areas for improvement must be identified so as to ensure that more inclusive, sensitive, and patient-centered care is (can be) provided to all individuals, regardless of their sexual orientation or gender identity. This research highlights important areas for improvement, such as the need for a physician to use gender-neutral language, avoid making assumptions, and recognize that certain medical conditions and behavioral patterns are more common among LGBTQ+ individuals, since this can have a direct or indirect effect on their gynecological health. This could include implementing targeted training modules for OBGYNs on inclusive communication, expanding LGBTQ+ health content in medical education curricula, and integrating pronoun and identity fields into electronic health records. Moreover, the results of our study emphasize the need for the OBGYN to create a safe environment for the patient to openly disclose his/her gender and sexual identity and discuss his/her sexual practices, enabling the OBGYN to provide more personalized care for each patient. By doing so, the risk of certain gynecological health issues may potentially be reduced.

Additional extended research is required to evaluate the impact that negative and the positive experiences during OBGYN appointments may have on the mental health and general well-being of LGBTQ+ patients. Such research is essential in order to fully summarize the importance of this topic for the LGBTQ+ community and to create clearer guidelines for healthcare professionals regarding optimal LGBTQ+ care in the field of obstetrics and gynecology. Moreover, research on the quality of obstetrical care of LGBTQ+ individuals is critically needed worldwide.

## Figures and Tables

**Table 1:** Pronouns of heterosexual and LGBTQ+ respondents. * Other includes “he/she/they”, “he/him”, “xe/him”, and “N/A”.

Pronouns	Heterosexual	LGBTQ+
She/her	115 (90.6%)	128 (99.2%)
She/they	4 (3.1%)	0 (0.0%)
They/them	4 (3.1%)	0 (0.0%)
Other *	4 (3.1%)	1 (0.8%)

**Table 2:** Reasons for not choosing to use female barrier contraception after trying them.

Reasons	Heterosexual	LGBTQ+
Discomfort	2 (28.6%)	1 (33.3%)
High price	1 (14.3%)	2 (66.7%)
Not necessary	2 (28.6%)	-
N/A	2 (28.6%)	-

**Table 3:** Reasons for the decision not to use female barrier contraception by the respondents who have not tried them.

Reasons	Heterosexual	LGBTQ+
Discomfort	47 (51.6%)	31 (41.3%)
High price	8 (8.8%)	13 (17.3%)
Not necessary	18 (19.8%)	21 (28%)
Not enough information	3 (3.3%)	1 (1.3%)
Uses other contraceptives	9 (9.9%)	1 (1.3%)
Feel ashamed	1 (1%)	4 (5.3%)
N/A	11 (12%)	11 (14.7%)

**Table 4:** Frequency of OBGYN visits among LGBTQ+ and heterosexual respondents.

Frequency of OBGYN Visits	Heterosexual	LGBTQ+	*p*-Value
At the time of completing the survey, did not visit an OBGYN regularly	10 (8.8%)	25 (22.5%)	0.0044
Less than once in 3 years	7 (6.1%)	10 (9.0%)	0.2391
1 time in 2–3 years	28 (24.6%)	33 (29.7%)	0.0925
1 time per year	43 (37.7%)	25 (22.5%)	0.0789
2–3 times per year	17 (14.9%)	9 (8.1%)	0.2404
1 time per half-year	5 (4.4%)	7 (6.3%)	0.3473
2–3 times per half-year	4 (3.5%)	2 (1.8%)	0.5508

**Table 5:** Respondents’ reasons for visiting an OBGYN in the past 2 years.

Reasons	Heterosexual	LGBTQ+	*p*-Value
Preventive check-up	62 (54.4%)	49 (44.1%)	0.5378
Menstrual cycle disorders	40 (35%)	47 (42.3%)	0.1067
Ovarian cysts, endometrial and cervical polyps, fibroids	19 (16.6%)	26 (23.4%)	
Vulvovaginal infections (chlamydiosis, bacterial vaginosis (BV), gonorrhea, etc.)	18 (12.9%)	19 (17.1%)	0.5227
Candidiasis	10 (8.8%)	8 (7.2%)	0.8645
Polycystic Ovary Syndrome	1 (0.9%)	2 (1.8%)	0.4795
Urinary tract infections	9 (7.9%)	4 (3.6%)	0.2494
Endometriosis	2 (1.8%)	2 (1.8%)	0.8845
Other/no information	25 (21.9%)	5 (4.5%)	0.0006

**Table 6:** Smoking among LGBTQ+ and heterosexual respondents.

Smoking Habits	Heterosexual	LGBTQ+	*p*-Value
Smoking	44 (34.1%)	59 (46.5%)	0.0373
Non-smoking	83 (64.3%)	65 (51.2%)	0.0373
N/A	2 (1.6%)	3 (2.3%)	0.6515

**Table 7:** Smoking rates among LGBTQ+ and heterosexual respondents.

Smoking Frequency	Heterosexual	LGBTQ+	*p*-Value
Up to 5 cigarettes per week	2 (4.6%)	3 (5.1%)	0.8997
Up to 5 cigarettes per day	13 (29.6%)	35 (59.3%)	0.0027
5–10 cigarettes per day	8 (18.2%)	7 (11.7%)	0.3686
10–20 per day	8 (18.2%)	11 (18.8%)	0.9523
>20 per day	3 (6.8%)	1 (1.7%)	0.1831
Only smoking electronic cigarette	6 (13.6%)	1 (1.7%)	0.0172
N/A	4 (9%)	1 (1.7%)	

**Table 8:** Psychoactive substance use among LGBTQ+ and heterosexual respondents.

Consumption of Psychoactive Substances	Heterosexual	LGBTQ+	*p*-Value
Use psychoactive substances	9 (7%)	26 (20.5%)	0.0013
Do not use psychoactive substances	120 (93%)	98 (77.2%)	0.0013

**Table 9:** Frequencies of urinary tract infection.

Urinary Tract Infection	Heterosexual	LGBTQ+	*p*-Value
1 time in life	4 (5.8%)	3 (3.8%)	0.5425
Less than once a year	44 (64.7%)	64 (80%)	0.0368
1 time per year	9 (13.2%)	3 (3.8%)	0.0351
1 time per half-year	1 (1.5%)	3 (3.8%)	0.3941
2–3 times per year	8 (11.8%)	7 (8.8%)	0.5448
2–3 times per half-year	1 (1.5%)	0 (0%)	0.2764
1 time per month	1 (1.5%)	0 (0%)	0.2764

## Data Availability

Data are contained within the article or Supplementary Materials.

## Supplementary Materials

The following supporting information can be downloaded at https://www.mdpi.com/article/10.3390/medicina61071209/s1, Table S1: Gender and sexual identities of LGBTQ+ respondents. Table S2: Reasons for birth control pill usage. Table S3: Libido changes in LGBTQ and heterosexual respondents while using CCP. Table S4: Reasons for first OBGYN appointment for LGBTQ+ and heterosexuals. Table S5: Regular exercise habits of LGBTQ and heterosexual respondents. Table S6: Frequencies of regular exercise among LGBTQ and heterosexual respondents. Table S7: Incidence of urinary tract infections among LGBTQ+ and heterosexual respondents. Table S8: BMI of heterosexual and LGBTQ+ patients.

## Appendix A

### Appendix A.1. General Questions

1. Your age (open question)
2. The country you live in (open question)
3. Your height (open question)
4. Your weight (open question)
5. Do you smoke?
  - Yes
  - No
  - I don’t want to answer
6. If you do, how much do you smoke on average per day?
  - I don’t smoke
  - 1–2 cigarettes per day
  - 3–5 cigarettes per day
  - 5–10 cigarettes per day
  - 10–20 cigarettes per day
  - >20 cigarettes per day
  - Other
7. Do you drink alcohol?
  - Yes
  - No
  - I don’t want to answer
8. If you do, how often do you drink alcohol?
  - I don’t drink alcohol
  - 1 pint/cup/glass per month
  - 1 pint/cup/glass per two weeks
  - 1 pint/cup/glass per week
  - 1 pint/cup/glass every 2–3 days
  - 1 pint/cup/glass everyday
9. Do you use any narcotic drugs?
  - Yes
  - No
  - I don’t want to answer
10. If you do, how often do you use narcotic drugs?
  - I don’t use drugs
  - Once a year
  - Once in half a year
  - Once in three months
  - Once a month
  - Once every two weeks
  - Once a week
  - More than once a week
11. Do you exercise regularly?
  - Yes
  - No
12. If you do, how often do you exercise?
  - I don’t exercise regularly
  - Everyday
  - 4–5 times a week
  - 2–3 times a week
  - 1 time a week
13. Do you use anabolic steroids?
  - Yes
  - No
14. Have you ever visited an OBGYN (obstetrician-gynecologist)?
  - Yes
  - No

### Appendix A.2. Questions About Seaxuality

1. Do you identify yourself as a part of the LGBTQ+ community?
  - Yes
  - No
2. What are your pronouns?
  - He/him
  - She/her
  - They/them
  - Other

### Appendix A.3. Questions for Members of the LGBTQ+ Community

1. Which term(s) best describes you?
  - Homosexual
  - Bisexual
  - Non-binary
  - Pansexual
  - Trans
  - Other
2. Are you open about your sexual and/or gender identity in society?
  - Yes
  - No

### Appendix A.4. Questions for Those Who Have Not Visited an OBGYN (Obstetrician-Gynaecologist)

1. Do you identify yourself as a part of the LGBTQ+ community?
  - Yes
  - No
2. What are your pronouns?
  - He/him
  - She/her
  - They/them
  - Other
3. For what reasons have you never visited an OBGYN (obstetrician-gynaecologist)? (mark all that apply)
  - I didn’t see the point
  - I have heard that services in public institutions are inadequate/low quality, and services in private institutions are too expensive
  - I’ve heard too many negative reviews and I don’t want to be uncomfortable
  - I don’t want to be asked about or reveal my gender identity
  - I’m afraid it will hurt
  - I’m afraid there’s something wrong with my body and I don’t want anyone to see it
  - Other
4. What would help/encourage you to see an OBGYN (obstetrician-gynaecologist)? (open question)

### Appendix A.5. Questions About Sex Life

1. Do you regularly have sexual intercourse?
  - Yes
  - No
2. Do you currently have more than one sexual partner?
  - Yes
  - No
3. Who do you have sex with?
  - Currently, I do not have regular sex
  - Men
  - Women
  - Both
4. Have you ever had sex with a man?
  - Yes
  - No
5. Have you ever had sex with a woman?
  - Yes
  - No
6. Do you use sex toys?
  - Yes
  - No
7. Do you share sex toys with your partner(s)?
  - Yes
  - No
8. Do you use barrier birth control methods during sex, such as condoms?
  - Yes
  - No
9. What barrier birth control methods do you use?
  - Male condom
  - Female condom
  - Diaphragm
  - Birth Control Sponge
  - Other
10. Do you know what female barrier birth control methods are?
  - Yes
  - No
11. Do you know how to use female barrier birth control?
  - Yes
  - No
12. Have you ever used female barrier birth control?
  - Yes
  - No
13. Do you know where you can buy female barrier birth control?
  - Yes
  - No
14. Would you use female birth control? If no, for what reasons you would not use female barrier birth control?
  - I would use female birth control
  - Inconvenient to use
  - The product is too expensive
  - It’s shameful/make’s me or my partner(s) uncomfortable
  - Other

### Appendix A.6. Questions for Members of the LGBTQ+ Community About Visits to an OBGYN (Obstetrician-Gynaecologist)

1. When you first visited an OBGYN, did you consider yourself part of the LGBTQ+ community?
  - Yes
  - No
2. How old were you when you first visited a doctor obstetrician-gynecologist? (open question)
3. For what reasons did you first visit an OBGYN?
  - Prophylactically (to check is everything is fine)
  - For certain complaints
  - Other
4. How often do you visit an OBGYN?
  - Currently I do not regularly visit an OBGYN
  - Less than every 3 years
  - Once in 2–3 years
  - Once a year
  - 2–3 times a year
  - Once in half a year
  - 2–3 time in half a year
  - Other
5. For what reasons did you have to visit an OBGYN in the last 1–2 years?
  - Currently I do not visit an OBGYN
  - Prophylactically
  - Infectious diseases of the vulva/vagina (bacterial vaginosis, chlamydiosis, gonorrhea etc.)
  - Candidiasis
  - Genital condylomatosis
  - Urinary tract infection
  - Genital injuries
  - Menstrual disorders (painful/long/heavy periods)
  - Ovarian cysts/endometrial and cervical polyps/uterine fibroids
  - Other
6. Has the regularity of your visits to an OBGYN changed when you started considering yourself part of the LGBTQ+ community?
  - Yes
  - No
7. When you first visit a NEW OBGYN, do they usually inquire about your sexual orientation before asking other questions?
  - They always ask before asking other questions
  - They usually ask
  - They only ask after prompting
  - Never ask
  - Even after declaring your sexual orientation, the OBGYN denies it
8. When you first visit a NEW OBGYN, before asking other questions, do they usually ask you what pronouns (he, she, they, etc.) you use?
  - Always
  - Sometimes
  - Never
9. When you visited an OBGYN, has the doctor at least once asked you questions about sexual relations with a man/birth control measures, etc., without first asking about your sexual orientation (and deciding that if you are a woman, you do have sexual relations with men)?
  - Yes
  - No
10. When you visited an OBGYN, has the doctor at least once asked questions about sexual relations with a woman without first asking about your sexual orientation (and deciding that you do have sexual relations with women)?
  - Yes
  - No
11. Is your current doctor obstetrician-gynecologist (if you have one) or the doctor obstetrician-gynecologist you visited last know that you belong to the LGBTQ+ community?
  - Yes
  - No
12. If your OBGYN does not know that you belong to the LGBTQ+ community—what are the reasons? (open question)
13. If your OBGYN know that you belong to the LGBTQ+ community, have they asked additional questions that they would not normally ask (e.g., about protection measures when having sex with a woman or other related questions)?
  - My doctor doesn’t know I’m LGBTQ+
  - Yes, they have asked additional questions
  - No, they have not asked additional questions
14. Do you feel that the behavior/attitude of the OBGYN has changed after finding out thet you are part of the LGBTQ+ community?
  - My doctor doesn’t know I’m LGBTQ+
  - Yes
  - No
15. Has an OBGYN ever tried to convince you that you should have your own biological children at least once in your life?
  - Yes
  - No, never
16. Do you ever ask your OBGYN questions/advice related to same-sex sexual relations?
  - Yes
  - No
17. If you could fully explain your gender and sexual orientation to your OBGYN, would you want to ask questions related to same-sex relationships? If so, what topics would be the most relevant to you? (open question)
18. Did you find an OBGYN with whom you could openly talk about your gender and sexual orientation? If so, was it in a public or private institution?
  - No
  - Yes, in a public institution
  - Yes, in a private institution
19. How often do you hear negative feedback about a OBGYN’s inappropriate treatment, behavior/attitude towards patients, in your environment?
  - Often
  - Sometime
  - Never
20. Do you hear negative feedback about an OBGYN’s inappropriate treatment, behavior/attitude towards patients, after the doctor finds out that they belong to the LGBTQ+ community?
  - Often
  - Sometime
  - Never

### Appendix A.7. Questions About an OBGYN for Non-LGBTQ+ Community Members

1. How old were you when you first visited a doctor obstetrician-gynecologist? (open question)
2. For what reasons did you first visit an OBGYN?
  - Prophylactically (to check is everything is fine)
  - For certain complaints
  - Other
3. How often do you visit an OBGYN?
  - Currently I do not regularly visit an OBGYN
  - Less than every 3 years
  - Once in 2–3 years
  - Once a year
  - 2–3 times a year
  - Once in half a year
  - 2–3 time in half a year
  - Other
4. For what reasons did you have to visit an OBGYN in the last 1–2 years?
  - Currently I do not visit an OBGYN
  - Prophylactically
  - Infectious diseases of the vulva/vagina (bacterial vaginosis, chlamydiosis, gonorrhea etc.)
  - Candidiasis
  - Genital condylomatosis
  - Urinary tract infection
  - Genital injuries
  - Menstrual disorders (painful/long/heavy periods)
  - Ovarian cysts/endometrial and cervical polyps/uterine fibroids
  - Other
5. When you first visit a NEW OBGYN, do they usually inquire about your sexual orientation before asking other questions?
  - They always ask before asking other questions
  - They usually ask
  - They only ask after prompting
  - Never ask
  - Even after declaring your sexual orientation, the OBGYN denies it
6. When you first visit a NEW OBGYN, before asking other questions, do they usually ask you what pronouns (he, she, they, etc.) you use?
  - Always
  - Sometimes
  - Never
7. When you visited an OBGYN, has the doctor at least once asked you questions about sexual relations with a man/birth control measures, etc., without first asking about your sexual orientation (and deciding that if you are a woman, you do have sexual relations with men)?
  - Yes
  - No
8. When you visited an OBGYN, has the doctor at least once asked questions about sexual relations with a woman without first asking about your sexual orientation (and deciding that you do have sexual relations with women)?
  - Yes
  - No
9. Has an OBGYN ever tried to convince you that you should have your own biological children at least once in your life?
  - Yes
  - No
10. How often do you hear negative feedback about OBGYN’s inappropriate treatment, behavior/attitude towards patients, in your environment?
  - Often
  - Sometime
  - Never

### Appendix A.8. Medical Questions

1. Do you currently have any gynecological complaints?
  - Yes
  - No
2. How often do you get urinary tract infections in your life?
  - I have never had a urinary tract infection
  - Less than once a year
  - Once a year
  - 2–3 times a year
  - Once in half a year
  - Once a month
  - Other
3. Have you ever been diagnosed with a sexually transmitted infection (chlamydia, gonorrhea, syphilis, HIV, herpes, etc.)
  - Yes
  - No
4. Have you ever been pregnant?
  - Yes
  - No
5. Have you given birth?
  - Yes
  - No
6. Have you ever had a medical or instrumental abortion?
  - No, never
  - Yes, 1 time
  - Yes, 2 times
  - Yes, 3 times
  - Yes, >3 times
7. Would you like to have biological children in the future?
  - Yes
  - No
  - I don’t know/never thought about it
  - I already have biological child(ren)
8. Do you have any non-gynecological/chronic diseases?
  - I don’t have any non-gynecological/chronic diseases
  - Depression
  - Hypothyroidism
  - Diabetes
  - Other
9. Are you currently taking any medications? What are the medications? (open question)
10. Are you currently using COCs (combined oral contraceptive pills)?
  - Yes
  - No
11. For what reasons do you use COCs (combined oral contraceptive pills)?
  - I don’t use COCs
  - To prevent pregnancy
  - To regulate the menstrual cycle
  - To prevent pregnancy and to regulate the menstrual cycle
  - Other
12. How has your libido changed since you started taking COCs (combined oral contraceptive pills)?
  - I don’t use COCs
  - Increased
  - Decreased
  - Didn’t change
13. What else would you like to add (notes, suggestions, observations)? (open question)
