# Supplementary material for: Characteristics of LGBTQ+ Patients and Their Care in Comparison with Heterosexual Individuals: What Is Important for the OBGYN?
Source: Medicina (Kaunas). 2025 Jul 2;61(7):1209. doi: 10.3390/medicina61071209 (PMC12298139; doi:10.3390/medicina61071209)
Supplement: Supplementary file 1 [file medicina-61-01209-s001.zip › Table S1. Sexual identity.pdf]

Sexual identity	Gender identity	Total number of respondents	Publicly declaring their identity	Publicly not declaring their identity	P value
Asexual	Cis-gender	4 (3.1%)	2 (3.4%)	2 (2.9%)	0.8852
Asexual	Non-binary	1 (0.8%)	1 (1.7%)	-	0.2811
Bisexual	Cis-gender	53 (41.7%)	21 (35.6%)	32 (47.1%)	0.1913
Bisexual/pasnexual	-	4 (3.1%)	-	4 (5.9%)	0.0584
Bisexual	Non-binary	1 (0.8%)	-	1 (1.5%)	0.3497
Bisexual	Non-binary/trans	1 (0.8%)	-	1 (1.5%)	0.3497
Homosexual	Cis-gender	38 (29.9%)	23 (39%)	15 (22%)	0.0378
Homosexual	Non-binary	2 (1.6%)	2 (3.4%)	-	0.1259
Pansexual	Cis-gender	14 (11%)	7 (11.8%)	7 (10.3%)	0.7781
Pansexual	Non-binary	3 (2.4%)	-	3 (4.4%)	0.1025
Pansexual	Non-binary/trans	1 (0.8%)	1 (1.7%)	-	0.2811
-	Non-binary	3 (2.4%)	2 (3.4%)	1 (1.5%)	0.4775
Queer	Queer	2 (1.6%)	-	2 (2.9%)	0.1842
