# Supplementary material for: Characteristics of LGBTQ+ Patients and Their Care in Comparison with Heterosexual Individuals: What Is Important for the OBGYN?
Source: Medicina (Kaunas). 2025 Jul 2;61(7):1209. doi: 10.3390/medicina61071209 (PMC12298139; doi:10.3390/medicina61071209)
Supplement: Supplementary file 1 [file medicina-61-01209-s001.zip › Table S2. Reasons for birth control pill usage.pdf]

Reasons for birth control pill usage	Heterosexual	LGBTQ+	p value
Pregnancy prevention	15 (75%)	4 (30.8%)	0.1882
Menstrual cycle regulation	12 (60%)	9 (69.2%)	0.1742
Endometriosis	1 (5%)	1 (7.7%)	0.5750
Other	4 (20%)	1 (7.7%)	0.5454
