# Supplementary material for: Characteristics of LGBTQ+ Patients and Their Care in Comparison with Heterosexual Individuals: What Is Important for the OBGYN?
Source: Medicina (Kaunas). 2025 Jul 2;61(7):1209. doi: 10.3390/medicina61071209 (PMC12298139; doi:10.3390/medicina61071209)
Supplement: Supplementary file 1 [file medicina-61-01209-s001.zip › Table S3. Libido changes.pdf]

Libido changes	Heterosexual	LGBTQ+	p value
Decrease	17 (50%)	12 (57.1%)	0.6062
Increase	1 (3%)	3 (14.3%)	0.1155
Didin't change	16 (47%)	6 (28.6%)	0.1739
