# Supplementary material for: Characteristics of LGBTQ+ Patients and Their Care in Comparison with Heterosexual Individuals: What Is Important for the OBGYN?
Source: Medicina (Kaunas). 2025 Jul 2;61(7):1209. doi: 10.3390/medicina61071209 (PMC12298139; doi:10.3390/medicina61071209)
Supplement: Supplementary file 1 [file medicina-61-01209-s001.zip › Table S4. Reasons for the first OBGYN appointment.pdf]

Reasons for the first OBGYN appointment	Heterosexual	LGBTQ+	p value
Due to certain complaints	72 (63.2%)	67 (60.4%)	0.6659
Prophylactically	42 (36.8%)	44 (39.6%)	0.6659
