# Supplementary material for: Characteristics of LGBTQ+ Patients and Their Care in Comparison with Heterosexual Individuals: What Is Important for the OBGYN?
Source: Medicina (Kaunas). 2025 Jul 2;61(7):1209. doi: 10.3390/medicina61071209 (PMC12298139; doi:10.3390/medicina61071209)
Supplement: Supplementary file 1 [file medicina-61-01209-s001.zip › Table S5. Physical activity.pdf]

Physical activity	Heterosexual	LGBTQ+	p value
Exercises regularly	47 (36.4%)	55 (43.3%)	0.2614
Don‘t exercise regularly	82 (63.6%)	72 (56.7%)	0.2614
