# Supplementary material for: Characteristics of LGBTQ+ Patients and Their Care in Comparison with Heterosexual Individuals: What Is Important for the OBGYN?
Source: Medicina (Kaunas). 2025 Jul 2;61(7):1209. doi: 10.3390/medicina61071209 (PMC12298139; doi:10.3390/medicina61071209)
Supplement: Supplementary file 1 [file medicina-61-01209-s001.zip › Table S6. Frequency of regular sports.pdf]

Frequency of regular sports	Heterosexual	LGBTQ+	p value
1 time per 2 weeks	1 (2.1%)	-	0.2770
1 time per week	6 (12.8 %)	15 (27.3%)	0.0709
2-3 times pwe week	27 (57.4%)	28 (50.9%)	0.5091
4-5 times per week	13 (27.7%)	7 (12.7%)	0.0583
Everyday	-	4 (7.3%)	0.0593
N/A	-	1 (1.8%)	0.3529
