# Supplementary material for: Characteristics of LGBTQ+ Patients and Their Care in Comparison with Heterosexual Individuals: What Is Important for the OBGYN?
Source: Medicina (Kaunas). 2025 Jul 2;61(7):1209. doi: 10.3390/medicina61071209 (PMC12298139; doi:10.3390/medicina61071209)
Supplement: Supplementary file 1 [file medicina-61-01209-s001.zip › Table S7. Urinary tract infections.pdf]

Urinary tract infections	**Heterosexual**	**LGBTQ+**	**P value**
Had a UTI at least once in their life	68 (52.7%)	80 (63%)	0.0568
Have never had a UTI	61 (47.3%)	44 (34.6%)	0.0568
N/A	-	3 (2.4%)	0.0791
