# Supplementary material for: Characteristics of LGBTQ+ Patients and Their Care in Comparison with Heterosexual Individuals: What Is Important for the OBGYN?
Source: Medicina (Kaunas). 2025 Jul 2;61(7):1209. doi: 10.3390/medicina61071209 (PMC12298139; doi:10.3390/medicina61071209)
Supplement: Supplementary file 1 [file medicina-61-01209-s001.zip › Table S8. BMI.pdf]

BMI	In total	Heterosexual	LGBTQ+	P value
<18,5	20 (7.8%)	12 (9.3%)	8 (6.3%)	0.3517
18.5-24.9	156 (60.9%)	76 (58.9%)	80 (63%)	0.5038
25-29.9	49 (19.2%)	24 (18.6%)	25 (19.7%)	0.8261
>29.9	28 (10.9%)	16 (12.4%)	12 (9.4%)	0.4489
N/A	3 (1.2%)	1 (0.8%)	2 (1.6%)	0.5522
